# Rapid Maxillary Expansion Has a Beneficial Effect on the Ventilation in Children With Nasal Septal Deviation: A Computational Fluid Dynamics Study

**DOI:** 10.3389/fped.2021.718735

**Published:** 2022-02-10

**Authors:** Shuai Chen, Jingying Wang, Xun Xi, Yi Zhao, Hong Liu, Dongxu Liu

**Affiliations:** ^1^Department of Orthodontics, School and Hospital of Stomatology, Cheeloo College of Medicine, Shandong University & Shandong Key Laboratory of Oral Tissue Regeneration & Shandong Engineering Laboratory for Dental Materials and Oral Tissue Regeneration, Jinan, China; ^2^Institute of Thermodynamics and Fluid Mechanics, School of Energy and Power Engineering, Shandong University, Jinan, China

**Keywords:** maxillary transverse deficiency, nasal septal deviation (NSD), rapid maxillary expansion (RME), nasal resistance, nasal aerodynamics, computational fluid dynamics (CFD)

## Abstract

Nasal septal deviation (NSD) is one of the most common nasal diseases. Different from common clinical examination methods, computational fluid dynamics (CFD) can provide visual flow information of the nasal cavity. The dimension and volume of the nasal cavity are easily affected by rapid maxillary expansion (RME). The purpose of this study was to use CFD to evaluate the effect of RME on the aerodynamics of the nasal cavity in children with maxillary transverse deficiency and NSD. Computational fluid dynamics was implemented after 3D reconstruction based on the CBCT of 15 children who have completed RME treatment. After treatment, the volume increases in the nasal cavity, nasopharynx, oropharynx, and pharynx were not statistically significant. The wall shear stress of the nasal cavity after RME, 1.749 ± 0.673 Pa, was significantly lower than that before RME, 2.684 ± 0.919 Pa. Meanwhile, the maximal negative pressure in the pharyngeal airway during inspiration was smaller after RME (−31.058 Pa) than before (−48.204 Pa). This study suggests that RME has a beneficial effect on nasal ventilation. The nasal airflow became more symmetrical in the bilateral nasal cavity after RME. Pharyngeal resistance decreased with the reduction in nasal resistance and the increase in the volume of oropharynx after RME.

## Introduction

Nasal septal deviation (NSD) (1) is one of the most frequently encountered diseases in the rhinology clinic, with a 39.9% incidence in children (2). It can result in imbalanced airflow in the bilateral nasal cavity, hypertrophy of the turbinate, and increased nasal resistance (3–5). Increased nasal resistance may lead to a chronic mouth-breathing pattern, causing dentofacial deformities, such as bilateral maxillary crossbite and maxillary transverse deficiency, during the growth and development of the craniofacial complex (6). Furthermore, increased nasal resistance may be responsible for obstructive sleep apnea syndrome (OSAS) because the pharyngeal airway tends to collapse with greater negative pressure in the pharyngeal (7). Therefore, it is necessary to evaluate the changes in nasal airflow dynamics after rapid maxillary expansion (RME) in children with maxillary transverse deficiency and NSD.

Rapid maxillary expansion is a well-known orthodontic procedure used in growing patients to correct maxillary transverse deficiency by opening the midpalatal suture and moving maxilla laterally (8, 9). It has been demonstrated that RME can increase nasal volume, reduce nasal resistance, and improve nasal airway ventilation by many studies using two-dimensional (2D) cephalometric measurement (10), CBCT (11), rhinomanometry (12), PSG (13), acoustic rhinometry (14), and computational fluid dynamics (CFD) (15, 16). Some studies have reported that RME increases the length of the septum and corrects NSD during childhood (17, 18). These RME-related studies were designed to explore morphological changes of the nasal structure and the measurement of nasal resistance. At present, studies on NSD in children are mainly divided into three categories: One includes comparative studies on the effect of NSD on craniofacial growth in children (19, 20); another focuses on the effect of septoplasty on nasal function (21–23); and the other includes studies that discuss the effect of maxillary expansion on nasal anatomy (17). Considering that NSD may cause the patient to develop mouth-breathing patterns leading to high vault and maxillary transverse deficiency, RME may be an effective treatment (17). However, there has been no reports related to changes of nasal airflow dynamics after RME in children with NSD and maxillary transverse deficiency. Therefore, a detailed investigation of nasal airflow is beneficial for us to further understand the relationship between nasal structure and nasal aerodynamics, and it could provide an important theoretical basis for early orthopedic treatment.

Currently, CFD has been recognized as an appropriate tool for simulating the dynamics of airflow, as it enables us to visualize three-dimensional (3D) airflow characteristics (24–28). The objective of this study was to evaluate nasal aerodynamics in children with maxillary transverse deficiency and NSD after RME using CFD.

## Materials and Methods

### Subjects

The study was reviewed and approved by the Shandong University School of Stomatology Research Ethic Board (protocol number 20200802). All written informed consents were received from the patients. The sample size was calculated based on an α of 0.05 and a β of 0.2 to detect the difference of 242.66 Pa in maximum pressure between groups, with a 300.22-Pa estimated standard deviation (29). The power analysis indicated that a sample size of 13 was required. The sample of this study consisted of 15 (6 boys and 9 girls with a median age of 9.57 ± 1.51 years) children with maxillary transverse deficiency (30) and NSD (31) who were treated with RME. The degree of NSD depends on the angle of the line between the crista galli and the premaxilla and the line between the crista galli and the most prominent point of the septal bone or cartilage. Septal body asymmetry is defined as the width measured from the lateral aspect of the septal body to the septal bone or cartilage. The overall degree of septal deviation and septal body asymmetry were 4.56 ± 1.33° and 2.16 ± 0.32 mm, respectively (see Supplementary Table 1 for other anatomical measurement results). The exclusion criteria included (1) history of orthodontic or orthopedic treatment, (2) systemic diseases or any other severe craniofacial anomalies except maxillary transverse deficiency and nasal septum deviation, (3) temporomandibular joint disorders, and (4) no history of tonsillectomy. The expander was a Hyrax device that was bonded to the first deciduous molars or first premolars and the first permanent molars. Parents were instructed to turn the expansion screw 0.5 mm per day until the palatal cusps of the maxillary first molars contacted the buccal cusps of the mandibular first molars after 14 ± 2 days. The opening of the palatal suture was 4.13 ± 0.41 mm.

### CBCT Data Acquisition

CBCT scans were taken (which was not routine) with the patient awake in the supine position and the Frankfort horizontal plane vertical to the floor by the same operator using the same CBCT scanner (New Tom 5G, Verona, Italy; scan time: 10 s, slice thickness: 0.3 mm, 110 kV, 5 mA). During scanning, patients were guided to close their mouths with the maximum intercuspation and hold their breath after the end of expiration.

CBCT images were obtained before and after treatment as T1 data and T2 data, respectively, and were stored in the DICOM (Digital Imaging and Communications in Medicine) file format.

### Three-Dimensional Virtual Model Reconstruction

**Figure 1:**
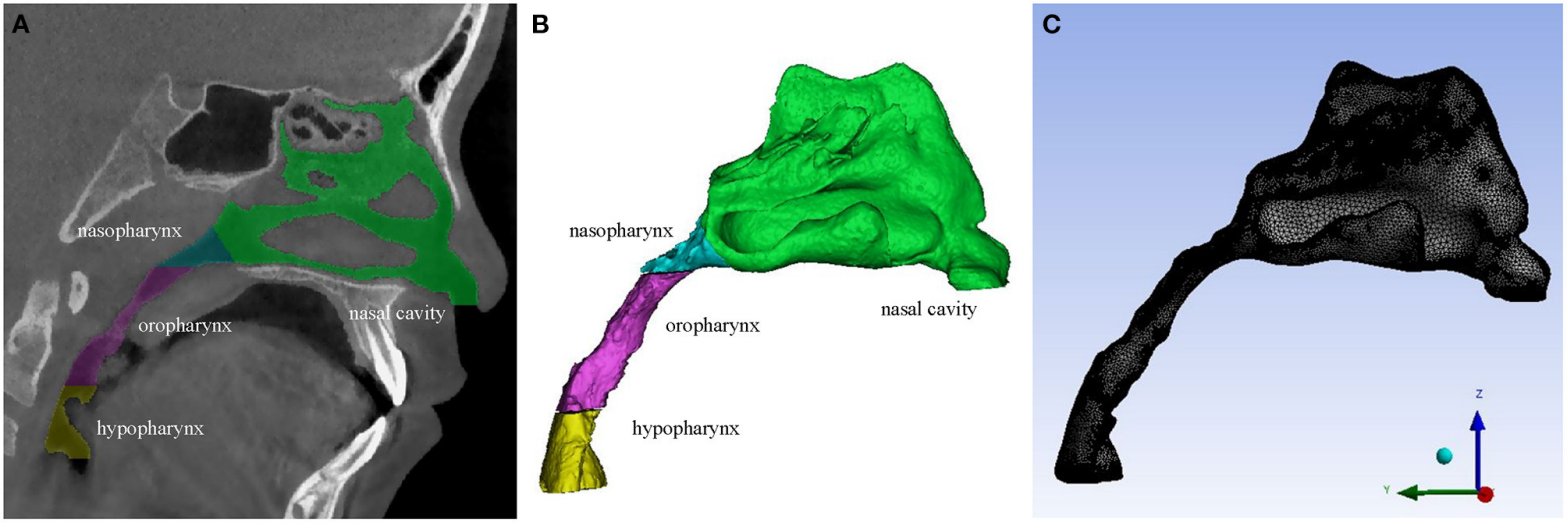
**(A)** A CBCT slice of the upper airway. **(B)** 3D model of the upper airway. **(C)** Mesh generation of the upper airway 3D geometry.

Three-dimensional models of the upper airway were constructed from the CBCT scans using the MIMICS 21.0 (Materialism's Interactive Medical Image Control System) software. The upper airway was isolated from other neighboring structures by setting the threshold of gray level between −1,024 Hounsfield Units (HU) and −220 HU. The sinuses were removed from the nasal cavity to simplify the 3D upper airway models in this study, according to Xiong et al.'s research (32). After reconstructing 2D segmented sets, primary smoothing of the models was performed to reduce computational cost and improve computational efficiency without affecting the main flow pattern inside (4). All 3D models were exported as stereolithography (STL) files (Figure 1).

### Mesh Generation

**Figure 2:**
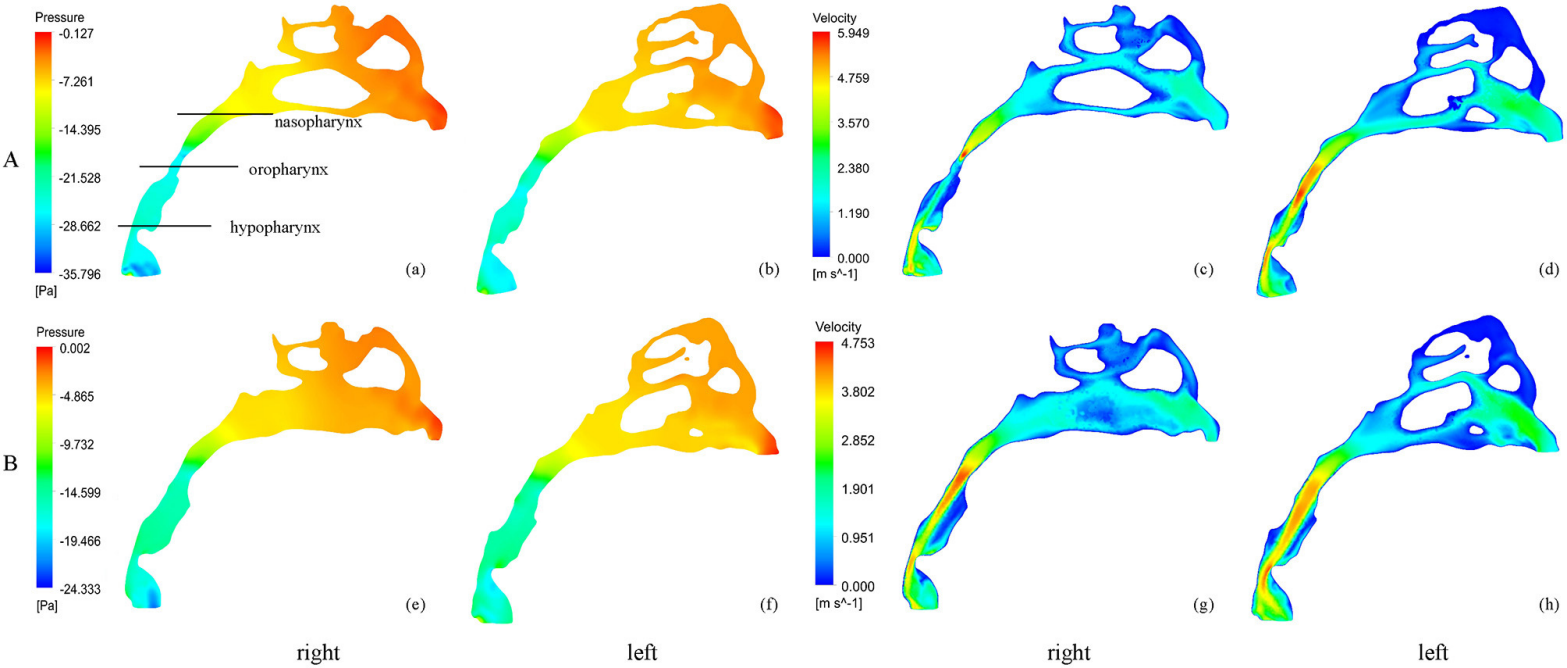
The pressure and velocity profile of the airflow before and after RME. **(A)** before RME, **(B)** after RME.

Firstly, five inflation layers were set at the wall to generate a finer grid near the wall. Then, the unstructured tetrahedral volumetric grids of the upper airway flow domain were generated (Figure 1). To compromise between the computational cost and accuracy, grid independence tests were performed by repeatedly solving the pressure drops of the nasopharynx, oropharynx, and hypopharynx planes (Figure 2) with four different element size meshes to establish grid independence solutions. The relative difference (RD) is defined as follows:


(1) $$\begin{array}{r} \text{RD}=|\frac{\text{the pressure drop of other grid numbers}-\text{the pressure drop of 1 million grids}}{\text{the pressure drop of 1 million grids}}|\times 100\% \end{array}$$

According to the comparison result, 530,000 grids were selected for subsequent calculations (Supplementary Table 2), resulting in a mesh of the pre- or post-RME model with at least 2.66 million elements. The maximum Skewness of the grids was 0.8. The 3D models were then loaded into the Reynolds Average Navier-Stokes CFD solver FLUENT v15.0 (ANSYS, Inc., Canonsburg, USA) for airflow simulation.

### Computational Fluid Dynamics

The inspiration flow in the upper airway was simulated using the Reynolds Average Navier-Stokes (RANS) CFD solver FLUENT v15.0, since the negative pressure resulting from inspiration is regarded as one of the causes of airway collapse in OSAS (33, 34). The air in the calculation domain was supposed to be a Newtonian, homogeneous, and incompressible fluid. The RANS equations were solved to account for the possible existence of turbulence with the standard k-ω model using the SIMPLE (semi-implicit method for pressure linked equation) method (35). For the inspiration process, a pressure condition (gauge pressure was 0 pa) was applied to the both nostrils (the flow outlet), and a negative velocity (V = Q/A, where Q was the volume flow rate of the model, and A was the area of the bottom of the hypopharynx) was applied to the bottom of the hypopharynx (the flow inlet). In this study, the constant inspired flow rate was set as 200 ml/s (16). For simplification, the factors of temperature, humidity and vibrissae were neglected in the simulation, while the gravity factor was considered. The direction of gravity is along the Y-axis as shown in Figure 1C. The calculation residual was set as 10^−6^. A second-order upwind scheme was used for discretizing the momentum, turbulent kinetic energy, and turbulent dissipation rate equations.

### Validation of CFD Model

In this paper, the wall static pressure results of three different CFD numerical models (Sp-Almaras, standard k-ε, standard k-ω, k-ω SST, and LES) were compared using an *in vitro* experimental system introduced in a previous study (36). Three pressure taps were set on the surface of the 1:1 scaled mechanical upper airway model, which were located in the nasopharynx, oropharynx, and hypopharynx, respectively (Supplementary Figure 1). The inspiratory experiment was carried out at a constant average flow rate of 650 ml/s. Computational fluid dynamics simulations were performed under the same airway geometry and boundary conditions. Regarding the wall pressure, the standard k-ω model yielded better agreement with the experimental data than the other CFD models (Supplementary Figure 2), with a maximum difference of <17%. The reason for this slightly larger RD may be due to the limitation of STL technology, which caused the small resin burrs inside the model to affect the air flow.

### CFD Outcome

Three planes were examined: (1) the choanae plane, a plane passing through the posterior nasal spine and the midpoint of the posterior upper edge of the nasal septum; (2) the palatal plane, a plane parallel to the hard palate connecting the anterior nasal spine and the posterior nasal spine; and (3) the superior border of the epiglottis plane, a plane parallel to Frankfort horizontal plane passing through the superior border of the epiglottis. The pharynx was further divided into three parts: nasopharynx, oropharynx, and hypopharynx. The volumes (Vol) of each section were measured.

After the simulation, all of the cross-sections from the nostril to choanae were selected along the Y-axis every 10 mm. Nasal resistance (*R* = Δ*P*/*Q*, the ratio between pressure changes from external nares to the choanae and total flow rate) (37), maximum velocity of the nasal cavity, and pressure drops (Δ*P* = *P*_max_-*P*_min_) were calculated from the outcome of the CFD analysis. Wall shear stress (wss), maximum negative pressure (P_min_), and maximum velocity (V_max_) of the pharynx were measured. Because the flow rate was constant in our study, Δ*P* reflected the changes in the resistance for each pharyngeal part.

### Statistical Analysis

Statistical analysis was performed by the SPSS (version 20.0, IBM, New York, USA) software package. Intra-examiner reliability was determined by performing the measurements for each CBCT image on two separate occasions by one examiner at a 2-week interval. The intraclass correlation coefficients were calculated, and then the mean of the 2 measurements was used in statistical analysis. The error of the method was calculated using the Dahlberg formula: ME= $\sqrt{\sum {\left(d\right)}^{2}/2n\;}$(where *d* indicates deviations between the two measurements, and n indicates the number of paired objects). All measured variables were described using the mean and the standard deviation. For normal distribution data, a paired *t*-test was used to compare the difference between T1 and T2. In the case of abnormal distributions, Wilcoxon signed rank test was used for comparison. *P* < 0.05 indicated that the difference was significant.

## Results

**Figure 3:**
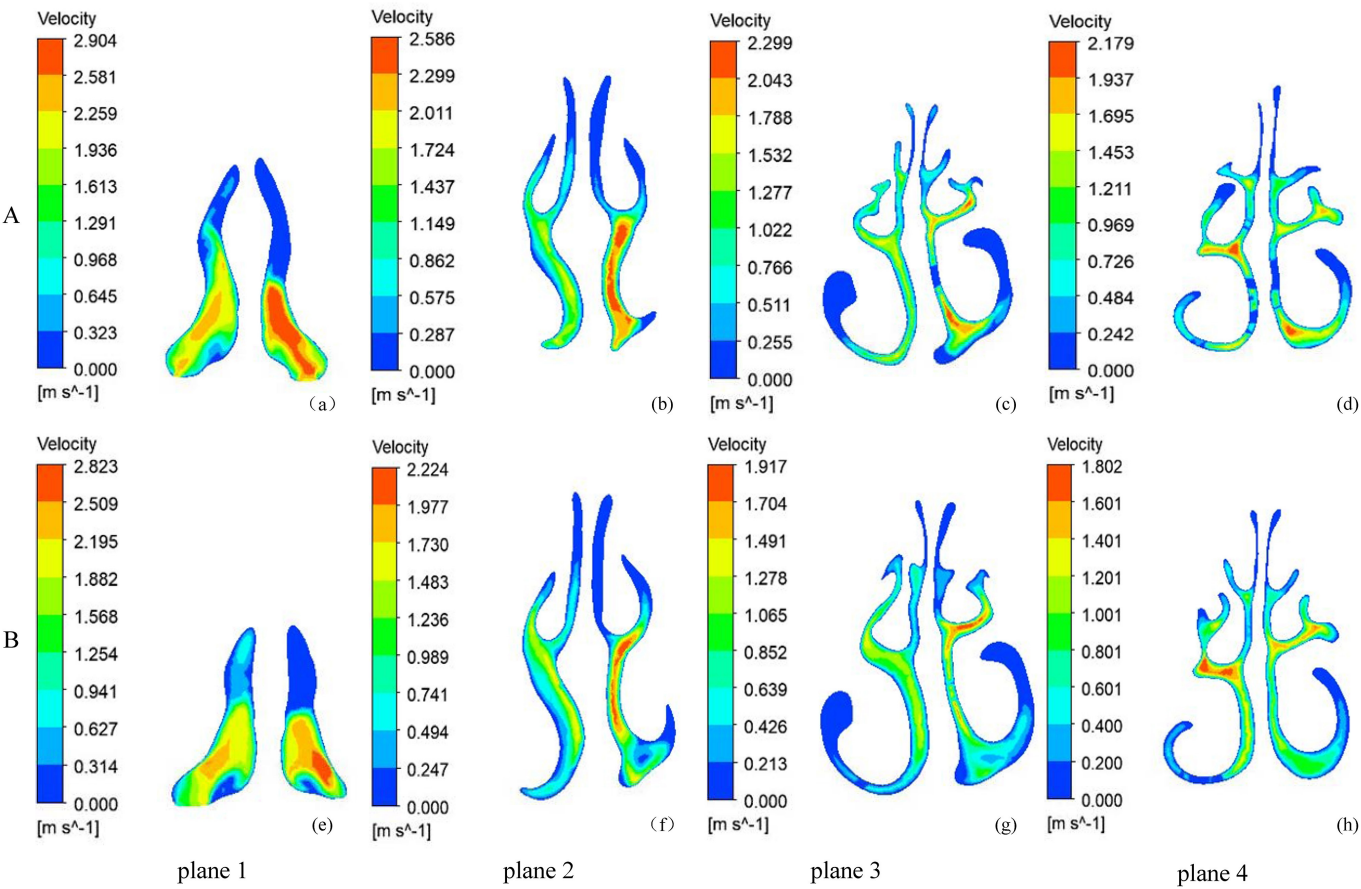
The velocity magnitude contours at five coronal sections of a typical subject with nasal septal deviation. **(A)** before RME, **(B)** after RME.

**Figure 4:**
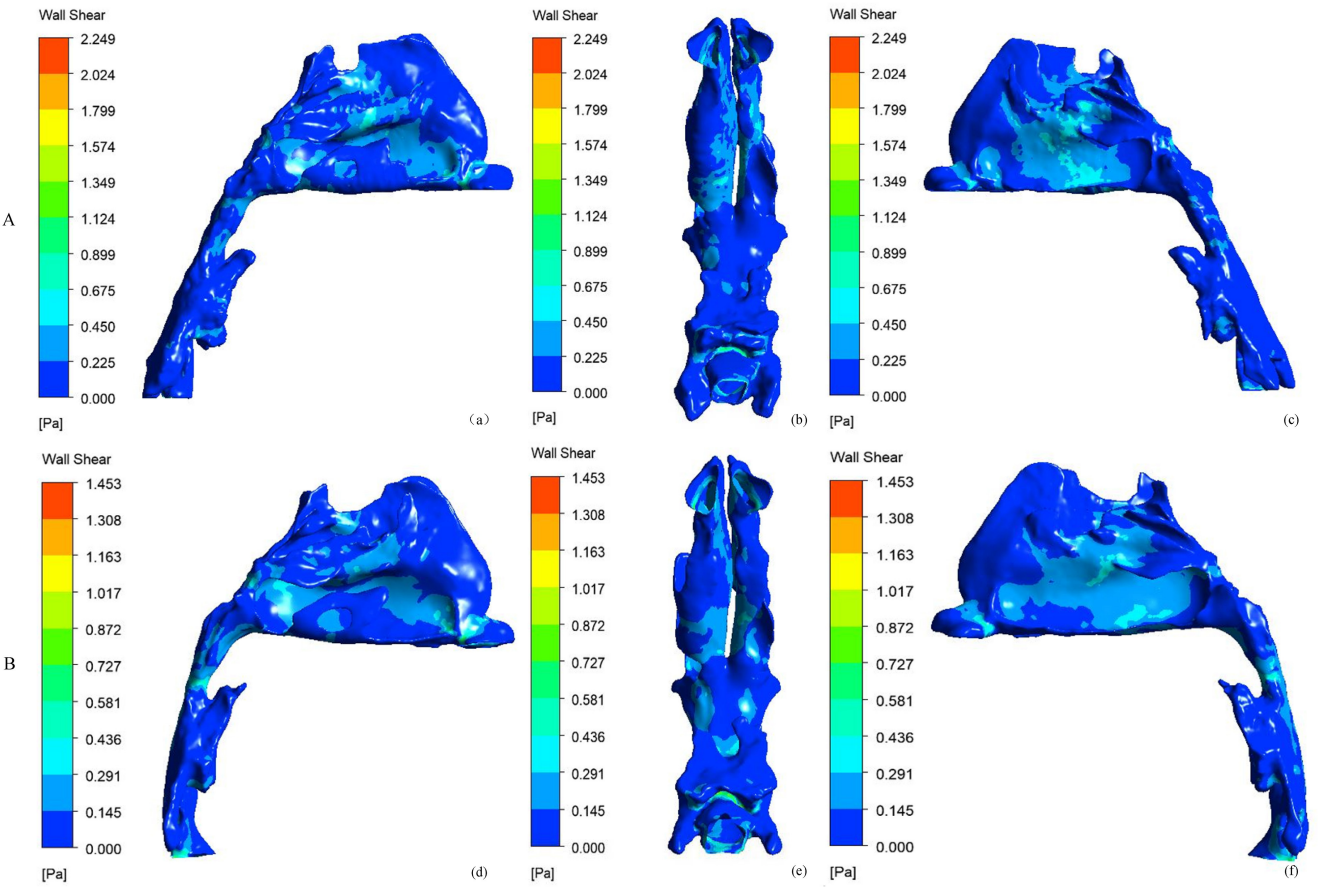
Side and bottom views of the wall shear stress (Pa) of nasal cavity. **(A)** before RME, **(B)** after RME.

*post-hoc* analysis showed that the average power (1-β) of all calculation results reached 0.84. The intraclass correlation coefficients for all measurements ranged from 0.94 to 0.97, indicating sufficient reliability. The ME ranged from 0.052 to 0.081 cm^3^ for volume measurements and from 0.42 to 0.95 Pa for maximal negative pressure. According to all repeated analyses, the measurement errors were negligible. Taking a 9-year-old girl as an example, Figures 1–**4** illustrate the results of RME.

### Volume of the Upper Airway

Table 1 shows that after treatment, the average increments of the nasal cavity, nasopharynx, oropharynx, and hypopharynx between T1 and T2 were 0.81, 0.41, 0.70, and 0.35 cm^3^, respectively (*P* < 0.05), indicating that RME did not significantly change the volume of the upper airway.

**Table 1:** Comparison between pre- and post-RME airway volume measurements.

**Volume (cm^**3**^)**	**Nasal** **cavity**	**Nasopharynx**	**Oropharynx**	**Hypopharynx**
T1	11.78 ± 1.00	3.42 ± 0.53	3.47 ± 1.28	2.76 ± 0.50
T2	12.60 ± 1.19	3.83 ± 0.67	4.17 ± 0.62	3.11 ± 0.35
*P*	0.086	0.052	0.074	0.052

### Velocity of the Nasal Cavity

Figure 3 shows the velocity magnitude contours at the coronal cross-sections in the subject. Before RME, the nasal airflow was obviously asymmetrical between the two sides of the NSD nose. Before RME, the major airflow passed through the middle nasal airway (between the inferior and middle turbinates around the septum) on the convex side, while it passed through the superior part of the nasal airway (between the middle and superior turbinates around the septum) in the concave side. After RME, the main airflow path was similar to that before the treatment, but the flow of the nasal cavity on both sides becomes relatively symmetrical. The maximal airflow velocity after RME, 4.754 m/s, was lower than that before RME, 5.990 m/s.

### Nasal Resistance

Nasal resistance after RME, 0.043 ± 0.017 Pa/(ml/s), was significantly smaller than that before RME, 0.073 ± 0.040 Pa/(ml/s) (Table 2; Figure 2). Before RME, the pressure drop was more drastic from the nostril to the choanae (Figure 2A). After RME, the pressure decreased smoothly along the airway from the nostril to the choanae (Figure 2B).

**Table 2:** The nasal and pharyngeal aerodynamic parameters from CFD simulation.

**Variables**		**T1**	**T2**	***P***
NR (Pa/(ml/s)	Mean	0.073	0.043	0.002^*^
	SD	0.040	0.017	
WSS_max_ (pa)	mean	2.684	1.749	0.008^*^
	SD	0.919	0.673	
V_max_ (m/s)	Mean	5.990	4.754	0.067
	SD	0.849	0.266	
P_min_ (pa)	Mean	−48.204	−31.058	0.004^*^
	SD	9.380	5.962	
Na-ΔP (pa)	Mean	6.967	4.478	0.016^*^
	SD	3.373	2.816	
Or-ΔP (pa)	Mean	12.841	10.584	0.226
	SD	3.633	1.721	
Hy-ΔP (pa)	Mean	17.746	14.482	0.080
	SD	3.143	1.757	

*NR, the nasal resistance; WSS_max_, the maximal wall shear stress in the nasal cavity; V_max_, the maximal velocity; P_min_, the maximal negative pressure in the pharynx; ΔP, pressure drop in the pharynx; Na, nasopharynx; Or, oropharynx; Hy, hypopharynx*.

* *Indicates a statistical significance at P < 0.05*.

### Wall Shear Stress of the Nasal Cavity

Figure 4 shows the side and bottom views of wall shear stress distribution of the nasal cavity. The maximal wall shear stress of the nasal cavity decreased from 2.684 ± 0.919 to 1.749 ± 0.673 Pa after RME. Before RME, a relatively larger shear stress was found in the middle and inferior region of the common meatus in the right nasal cavity (Figure 4A). After RME, the wall shear stress of the nasal cavity decreased significantly, and a relatively larger shear stress was found in the middle region of the common meatus in both sides of the nasal cavity (Figure 4B).

### Pressure in the Pharynx

The minimal negative pressure in the pharynx and nasopharynx drop after RME was significantly smaller than that before RME, whereas there were no significant changes in the oropharynx and hypopharynx pressure drop (Table 2; Figure 2).

## Discussion

We investigated the changes of nasal airflow in children with NSD and maxillary transverse deficiency. After RME, nasal airflow became more symmetric and was observed in the middle and inferior region of the common meatus, which is beneficial for heating the inspire airflow, as the inferior turbinates are the main region of heat exchange from mucosa (38). The velocity of the nasal cavity was decreased, showing that the inspired airflow had enough time to contact the nasal mucosa to facilitate heating/cooling and humidification of inspired air.

The wall shear stresses was the vital factor in controlling the defense mechanisms of the upper airway (39). Excessive wall shear stresses related to a greater velocity of airflow along the wall of the nasal cavity may cause irritation of the blood vessels (40) and adversely affect the functioning of epithelial cells (41). The greater wall shear stress in the nasal cavity was decreased after RME, which may play a protective role in the mucous membrane of the nasal cavity.

Before RME, the pressure of pharyngeal decreased sharply, and the pressure drop increased due to increased nasal resistance. After treatment, the maximal negative pressure and the resistance of the pharyngeal during inspiration decreased with decreased nasal resistance and increased oropharyngeal volume after RME. Likewise, it was demonstrated in a study by Iwasaki et al. (37) and Hur et al. (42). The decreased resistance of the pharyngeal (pressure drop) after RME may cause a decrease in pharyngeal compliance and an increase in the capability to prevent pharyngeal collapse. This mechanism may relieve the symptoms of OSAS in children.

This research noted volume increases in the nasal cavity (0.81 cm^3^) and oropharynx (0.70 cm^3^). The increase in the oropharyngeal volume may be because the tongue is moved forward with the increase in the oral volume after RME (43). The measurement of the volume of the nasopharynx (0.41 cm^3^) showed a smaller increase than that of the nasal cavity, as circummaxillary structures, such as the zygomatic bone and sphenoid bone, are more resistant to RME (44). The change in the volume of the hypopharynx (0.35 cm^3^) may be due to the young growth of the mandible, which can promote the sagittal growth of the mandible and increase the volume of the airway (45, 46). These findings suggested that nasal and pharyngeal airflow characteristics responded positively to RME. Rapid maxillary expansion may be an effective option for children with NSD and maxillary transverse deficiency.

There are still some limitations to this study. Firstly, the sample size was relatively small. Secondly, the interaction between the tissues around the upper airway and the airflow was not taken into account. Thirdly, there was a lack of comparison between traditional RME and bone-borne RME. The latter is related to greater skeletal expansion (47), which may have a greater impact on airflow in the nasal cavity. In the future, we will conduct studies to examine the effects of RME and bone-borne RME on nasal aerodynamics in patients with different locations of septal deviation. We will also investigate different extents of inferior turbinate hypertrophy and further study the fluid–solid interaction model of the upper airway to increase the validity of the results.

## Data Availability Statement

The original contributions presented in the article/Supplementary Material, further inquiries can be directed to the corresponding author.

## Ethics Statement

The studies involving human participants were reviewed and approved by School of Stomatology Shandong University Research Ethic Board. Written informed consent to participate in this study was provided by the participants' legal guardian/next of kin.

## Author Contributions

SC contributed to conception, design, data acquisition, analysis, interpretation, and drafted and critically revised the manuscript. JW contributed to analysis and interpretation and critically revised the manuscript. XX contributed to data acquisition and analysis. YZ and HL contributed to data acquisition. DL contributed to conception, design, interpretation, and drafted and critically revised the manuscript. All authors gave final approval and agree to be accountable for all aspects of the work.

## Funding

This work was supported by the Clinical Research Center of Shandong University (Grant Number 2020SDUCRCA005) and Shandong University Postgraduate Education and Teaching Excellence Cultivation Program of China (Grant Number ZY2019004).

## Conflict of Interest

The authors declare that the research was conducted in the absence of any commercial or financial relationships that could be construed as a potential conflict of interest.

## Publisher's Note

All claims expressed in this article are solely those of the authors and do not necessarily represent those of their affiliated organizations, or those of the publisher, the editors and the reviewers. Any product that may be evaluated in this article, or claim that may be made by its manufacturer, is not guaranteed or endorsed by the publisher.

## Supplementary Material

Supplementary Figure 1 Experimental airway model with pressure taps locations.

Supplementary Figure 2 Comparison of wall static pressure between experiments and three different CFD models.

Supplementary Table 1 Statistical comparisons of morphological measurement.

Supplementary Table 2 Grid independence test.

Supplementary Table 3 Changes of airway volume and aerodynamic parameters before and after rapid maxillary expansion.
