# Supplementary figures and images for: Rapid Maxillary Expansion Has a Beneficial Effect on the Ventilation in Children With Nasal Septal Deviation: A Computational Fluid Dynamics Study
Source: Front Pediatr. 2022 Feb 10;9:718735. doi: 10.3389/fped.2021.718735 (PMC8866691; doi:10.3389/fped.2021.718735)

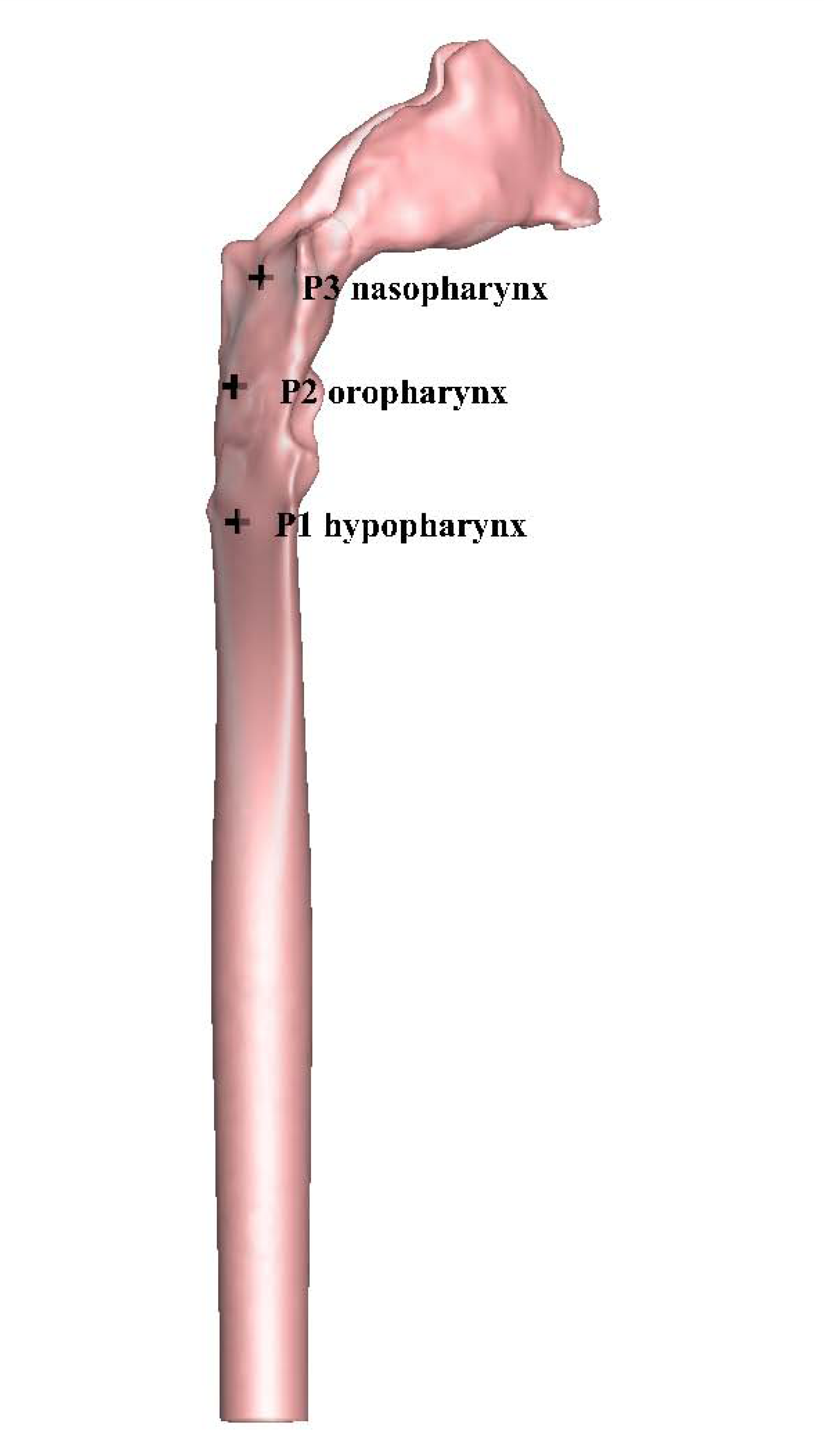

Supplement: Supplementary Figure 1 — Experimental airway model with pressure taps locations. [file Image_1.TIFF]

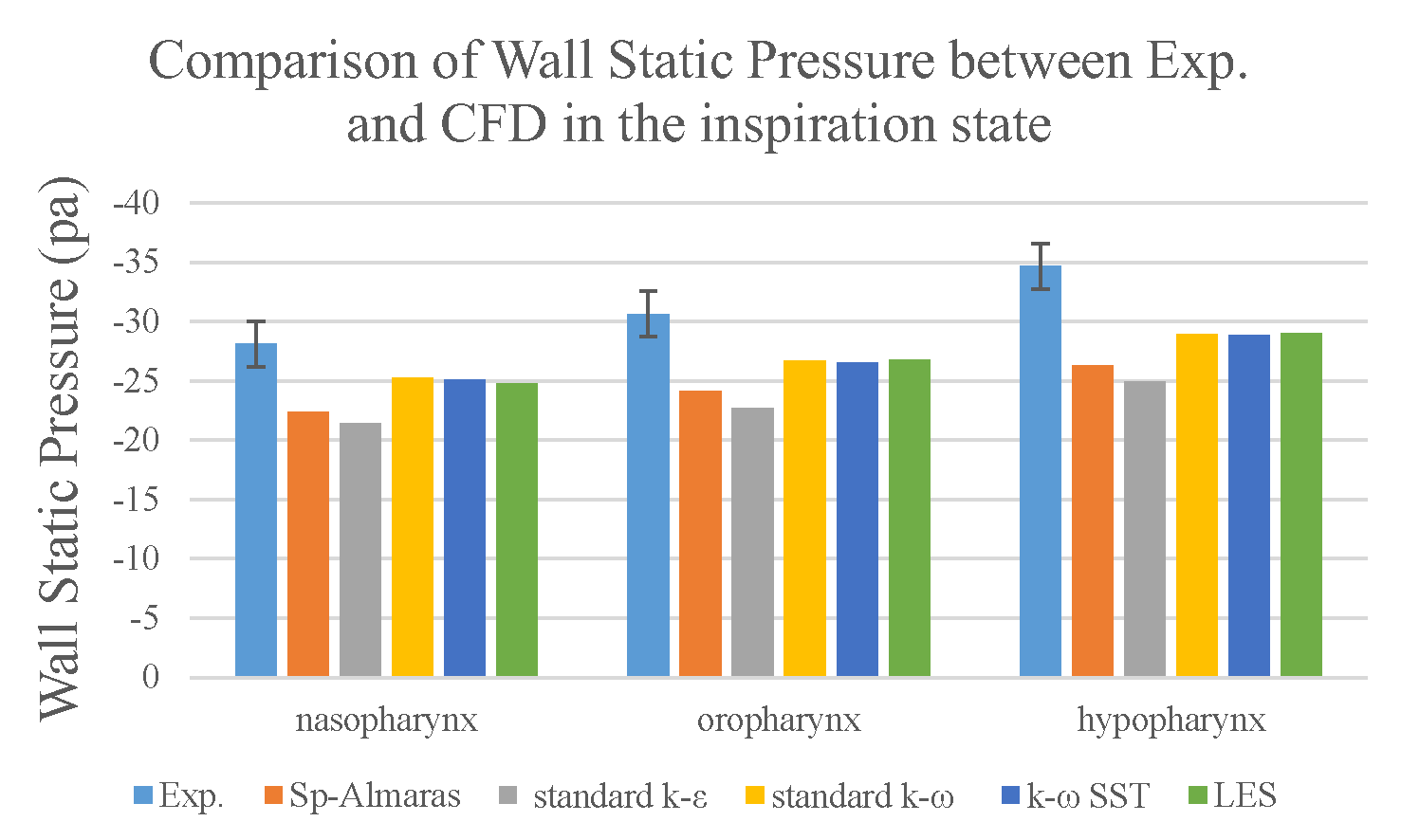

Supplement: Supplementary Figure 2 — Comparison of wall static pressure between experiments and three different CFD models. [file Image_2.TIFF]
