# Supplementary material for: Rapid Maxillary Expansion Has a Beneficial Effect on the Ventilation in Children With Nasal Septal Deviation: A Computational Fluid Dynamics Study
Source: Front Pediatr. 2022 Feb 10;9:718735. doi: 10.3389/fped.2021.718735 (PMC8866691; doi:10.3389/fped.2021.718735)
Supplement: Supplementary Table 1 — Statistical comparisons of morphological measurement. [file Table_1.DOCX]

Table S1. Statistical comparisons of morphological measurement

| Variables | nasal width(mm) | SNA(°) | SNB(°) | ANB(°) |
| --- | --- | --- | --- | --- |
| T1  T2 | 26.78±0.98  29.54±1.49 | 78.40±0.57  80.65±0.92 | 80.82±0.45  79.94±0.63 | -2.42±0.11  0.95±0.64 |
| P | 0.017 | 0.070 | 0.090 | 0.024 |
