# Supplementary material for: Rapid Maxillary Expansion Has a Beneficial Effect on the Ventilation in Children With Nasal Septal Deviation: A Computational Fluid Dynamics Study
Source: Front Pediatr. 2022 Feb 10;9:718735. doi: 10.3389/fped.2021.718735 (PMC8866691; doi:10.3389/fped.2021.718735)
Supplement: Supplementary Table 2 — Grid independence test. [file Table_2.DOCX]

Table S2. Grid independence test

| Flow variables | plane | Relative difference (%) | | | |
| --- | --- | --- | --- | --- | --- |
|  |  | 300000 grids | 530000  grids | 810000  grids | 1000000  grids |
| Pressure  drop | nasopharynx | 6.72 | 2.02 | 1.54 | / |
|  | oropharynx | 6.24 | 2.08 | 1.44 | / |
|  | hypopharynx | 6.45 | 2.08 | 1.65 | / |
