# Supplementary material for: Rapid Maxillary Expansion Has a Beneficial Effect on the Ventilation in Children With Nasal Septal Deviation: A Computational Fluid Dynamics Study
Source: Front Pediatr. 2022 Feb 10;9:718735. doi: 10.3389/fped.2021.718735 (PMC8866691; doi:10.3389/fped.2021.718735)
Supplement: Supplementary Table 3 — Changes of airway volume and aerodynamic parameters before and after rapid maxillary expansion. [file Table_3.DOCX]

Table S3. Changes of airway volume and aerodynamic parameters before and after rapid maxillary expansion

|  | T1 | | | | | | | T2 | | | | | | |
| --- | --- | --- | --- | --- | --- | --- | --- | --- | --- | --- | --- | --- | --- | --- |
|  | volume | | | | CFD | | | volume | | | | CFD | | |
| patient | Nc(cm^3^) | Na(cm^3^) | Or(cm^3^) | Hy(cm^3^) | NR(Pa/(ml/s)) | WSS_max_(pa) | P_min_(pa) | Nc(cm^3^) | Na(cm^3^) | Or(cm^3^) | Hy(cm^3^) | NR(Pa/(ml/s)) | WSS_max_(pa) | P_min_(pa) |
| 1 | 11.49 | 3.90 | 5.81 | 3.05 | 0.08 | 3.30 | -44.74 | 13.05 | 3.53 | 4.86 | 2.83 | 0.04 | 1.90 | -28.89 |
| 2 | 11.46 | 3.10 | 2.36 | 2.12 | 0.11 | 3.72 | -53.88 | 11.53 | 3.58 | 4.24 | 2.93 | 0.06 | 3.23 | -33.97 |
| 3 | 11.33 | 4.49 | 2.19 | 2.30 | 0.02 | 1.39 | -30.97 | 12.32 | 3.77 | 3.76 | 3.20 | 0.02 | 0.76 | -25.94 |
| 4 | 12.27 | 4.41 | 2.43 | 2.76 | 0.08 | 2.86 | -43.84 | 11.48 | 3.8 | 3.50 | 2.94 | 0.04 | 1.85 | -26.84 |
| 5 | 10.34 | 3.16 | 3.42 | 3.44 | 0.15 | 4.15 | -55.26 | 10.74 | 2.8 | 3.67 | 3.82 | 0.07 | 2.57 | -33.06 |
| 6 | 11.83 | 2.66 | 3.42 | 2.99 | 0.10 | 3.43 | -59.34 | 12.58 | 3.51 | 4.08 | 3.35 | 0.05 | 2.24 | -37.74 |
| 7 | 12.76 | 3.34 | 3.40 | 2.42 | 0.05 | 1.87 | -38.16 | 13.08 | 4.25 | 4.55 | 3.09 | 0.03 | 1.27 | -23.03 |
| 8 | 11.10 | 3.54 | 5.24 | 3.04 | 0.07 | 2.75 | -48.34 | 13.46 | 4.75 | 4.32 | 2.67 | 0.04 | 1.71 | -31.93 |
| 9 | 11.46 | 3.31 | 5.11 | 2.93 | 0.06 | 1.95 | -47.44 | 10.87 | 3.86 | 4.64 | 3.48 | 0.04 | 1.40 | -24.64 |
| 10 | 10.50 | 3.78 | 2.04 | 2.93 | 0.12 | 3.60 | -48.70 | 13.21 | 4.88 | 3.19 | 2.85 | 0.07 | 2.34 | -33.04 |
| 11 | 12.30 | 2.97 | 1.70 | 2.53 | 0.01 | 1.56 | -65.04 | 14.16 | 2.89 | 3.81 | 2.97 | 0.01 | 1.14 | -43.10 |
| 12 | 12.51 | 2.89 | 2.58 | 3.23 | 0.08 | 3.11 | -58.87 | 13.68 | 4.29 | 3.65 | 3.14 | 0.05 | 1.72 | -38.35 |
| 13 | 14.25 | 3.53 | 4.16 | 2.91 | 0.01 | 1.28 | -34.48 | 14.33 | 4.72 | 4.57 | 3.52 | 0.01 | 0.72 | -24.61 |
| 14 | 12.38 | 3.18 | 3.58 | 3.27 | 0.07 | 2.13 | -48.96 | 13.38 | 3.93 | 4.12 | 3.40 | 0.04 | 1.49 | -34.89 |
| 15 | 10.82 | 3.06 | 4.59 | 1.53 | 0.09 | 3.14 | -45.02 | 11.12 | 2.85 | 5.6 | 2.56 | 0.05 | 1.89 | -25.84 |

Nc: nasal cavity; Na: nasopharynx; Or: oropharynx; Hy: hypopharynx; NR, the nasal resistance; WSSmax, The maximal wall shear stress in nasal cavity; Pmin, the maximal negative pressure in the pharynx; CFD: aerodynamic parameters
